# Parenteral micronutrient supplementation enhances mammary immune function and colostrum–milk quality by modulating cytokine profiles and oxidative stress in transition crossbred cows

**DOI:** 10.3389/fimmu.2025.1669246

**Published:** 2025-09-12

**Authors:** Yallappa M. Somagond, Pravasini Das, Ajay Kumar Dang, Dhawal K. Yadav, Priyanka M. Kittur, Bibhudatta S.K. Panda, Pooja Devi, Aarti Kamboj, Mohanned Naif Alhussien

**Affiliations:** ^1^ Lactation and Immuno-Physiology Laboratory, ICAR-National Dairy Research Institute, Karnal, Haryana, India; ^2^ National Research Centre, Mithun, Medziphema, Nagaland, India; ^3^ Guru Angad Dev Veterinary and Animal Sciences University, Ludhiana, Punjab, India; ^4^ Reproductive Biotechnology, TUM School of Life Sciences, Technical University of Munich, Freising, Germany

**Keywords:** periparturient cattle, injectable trace mineral, mammary infection, inflammatory cytokines, phagocytic cells

## Abstract

**Background:**

The transition period in dairy cattle is marked by oxidative stress and immune suppression linked to altered micromineral status. This study evaluated whether parenteral supplementation with trace elements and vitamins could enhance mammary health and improve the immunonutritional quality of colostrum and milk.

**Methods:**

Twenty-four multiparous cross-bred cows were blocked by parity and projected yield, then assigned to control, multivitamin (MV; vitamins A, B-complex, D₃, E), multi-mineral (MM; Copper (Cu), Manganese (Mn), Selenium (Se), and Zinc (Zn)), or combined multivitamin and multi-mineral (MMMV) groups. Intramuscular injections were administered on days −30, −15, −7, 0, +7, +15, and +30 relative to calving. Longitudinal sampling was conducted on days 0, 2, 3, 4, 7, 15, and 30 postpartum.

**Results:**

MMMV cows produced colostrum and milk with higher fat and protein percentages, stable lactose, and greater concentrations of insulin-like growth factors and immunoglobulins than all other groups (P < 0.05). Mammary health indicators improved concomitantly: somatic cell counts fell, the neutrophil-to-macrophage ratio normalised, and phagocytic activity of both cell types increased. These functional improvements were accompanied by reduced expression of toll-like and chemokine receptors in milk phagocytes. Additionally, the cytokine profile shifted toward an anti-inflammatory state evidenced by lower levels of IL-1β, IL-6, IL-8, IL-17A, and IFN-γ, and higher levels of IL-4 and IL-10. Reduced oxidative stress was indicated by decreased activities of superoxide dismutase, catalase, and glutathione peroxidase in the milk whey of the MMMV group. The MM and MV treatments conferred intermediate benefits, whereas the control group showed the greatest inflammatory and oxidative stress.

**Conclusions:**

Repeated parenteral delivery of complementary trace minerals and vitamins throughout the transition period enhances mammary innate immunity, attenuates inflammation and oxidative stress, and augments the nutritive and immunological value of colostrum and milk. This approach offers a practical intervention to safeguard udder health and optimise passive immune transfer to calves.

## Graphical Abstract

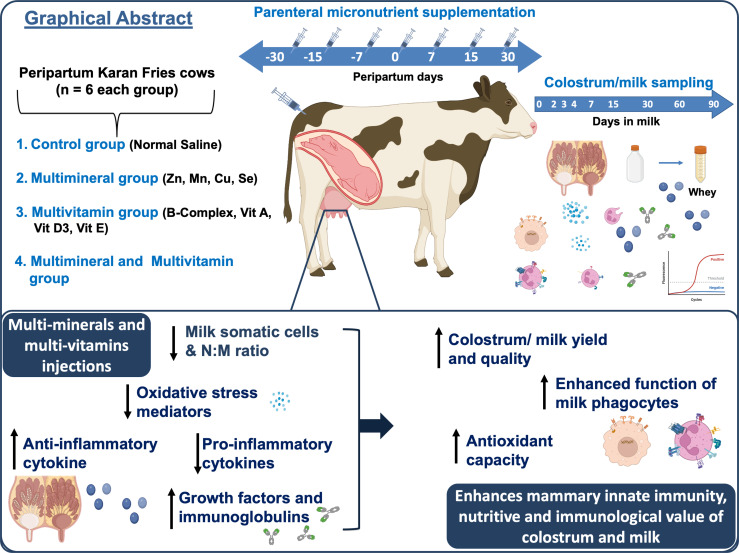

## 1 Introduction

The transition period in dairy cattle, spanning about three weeks before and after parturition, involves profound metabolic and immunological changes that heighten disease susceptibility (1). Reduced dry matter intake, negative energy balance, hormonal fluctuations, and oxidative stress impair immune competence (2, 3), predisposing cows to intramammary inflammation and affecting udder health and milk quality. At the same time, the mammary gland undergoes dynamic secretory changes, with colostrum and early milk enriched in immunoglobulins, cytokines, antioxidants, vitamins, minerals, and antimicrobial peptides, which are vital for neonatal immunity (4, 5). The composition and function of these secretions are shaped by maternal physiology and the activity of mammary-resident immune cells, particularly phagocytes, which orchestrate local defense and modulate cytokine balance (3, 6). Therefore, enhancing antioxidant defenses and supporting immune cell function during this period is critical for protecting mammary health and ensuring high-quality colostrum and milk, while also preparing cows to meet the increased nutritional demands of the transition phase.

To counter reduced feed intake and the simultaneous rise in antioxidant requirements during the periparturient period, supplementation with antioxidants, particularly vitamins and trace elements, is essential (7). Research has shown that low concentrations of trace elements and vitamins in blood during the periparturient period can compromise numerous immune functions and trigger various inflammatory conditions in cattle (8, 9). Essential vitamins (A, B12, D, and E) and minerals (such as copper, selenium, and iron) serve as cellular antioxidants and modulators of the immune system, playing a critical role in the health and productivity of dairy cows (8, 10). Administering these trace elements and vitamins to periparturient cows has been shown to significantly reduce the occurrence of parturition-related diseases, enhance the immune response, and facilitate a quicker return to homeostasis (9, 11). A major limitation of oral micronutrient supplementation in dairy cattle is reduced absorption due to ruminal degradation, microbial interactions, and the formation of insoluble complexes, all of which hinder bioavailability (12, 13). Despite recent innovations in oral micronutrient delivery, such as encapsulation and rumen-bypass formulations (14, 15), challenges with absorption remain. Consequently, injectable micronutrients have attracted attention as a promising alternative, offering more efficient and reliable delivery of essential nutrients during the transition period (16–19).

While the beneficial effects of oral multivitamin and multimineral supplementation on production performance and postpartum disease prevention have been well documented, the role of injectable antioxidant micronutrients in modulating immune responses in transition dairy cows remains insufficiently underexplored. In our previous work, we demonstrated that parenteral micronutrient supplementation during the transition period enhances systemic neutrophil function and reduces circulating inflammatory mediators in dairy cows (20). We hypothesized that supplementation would reduce mammary oxidative stress and inflammation, thereby enhancing mammary immunity and improving colostrum and milk quality. To test this hypothesis, the present study investigated organ-specific immunomodulatory effects within the mammary gland microenvironment. Specifically, we assessed the impact of parenteral vitamin and mineral administration on the functional activity of mammary-resident phagocytic cells, local inflammatory responses, and the bioactive composition of mammary secretions. This tissue-targeted approach aims to elucidate the mechanisms through which micronutrient supplementation influences mammary immune function and, consequently, affects the yield and immunological quality of colostrum and milk. Ultimately, these findings may inform nutritional strategies to enhance passive immunity transfer and productivity in periparturient dairy cattle.

## 2 Materials and methods

### 2.1 Ethical permission

The guidelines for animal experiments outlined by the Animal Ethics Committee of the ICAR-National Dairy Research Institute (NDRI), Karnal, India, according to article 13 of the CPCSEA rules, laid down by the Government of India, were followed during all the experiments. The animal study was reviewed and approved under order no. 42-IAEC-18-6.

### 2.2 Location of study and climatological conditions

The present study was conducted from August to March on 24 healthy peripartum Karan Fries (Holstein Friesian × Tharparkar) cows at the Livestock Research Centre (LRC), National Dairy Research Institute (NDRI), Karnal, India (29°43′ N, 77°2′ E; 245–250 m above mean sea level). The study site is located in the Indo-Gangetic alluvial plain and falls within a semi-arid zone, characterized by hot summers with temperatures up to 45 °C and cold winters with temperatures as low as 4 °C. The region receives about 70 cm of annual rainfall, and the relative humidity varies between 41% and 85%.

### 2.3 Animal selection and categorization for experimentation

Twenty-four healthy Karan Fries crossbred peripartum cows were randomly selected from the Livestock Research Centre (LRC), ICAR-NDRI, Karnal, India. The animals were divided into four groups of six cows each. Selection criteria included parity (3rd to 4th lactation), average body weight (420–450 kg), and body condition score (3.25–3.5), to minimize baseline variation among experimental groups. All selected cows were high-yielding (>10 kg/day) by Indian dairy standards, making them more susceptible to transition stress and intramammary infections during early lactation compared to local breeds. Throughout the experimental period, routine health monitoring was carried out to ensure that all animals remained free from physiological, pathological, or infectious disorders. The cows were housed individually in well-ventilated stalls and managed under uniform conditions to minimize environmental and handling stress. Approximately one week prior to the expected calving date, each cow was moved to a separate calving pen and remained there until 4–5 days postpartum for close observation and care.

**Figure 1:**
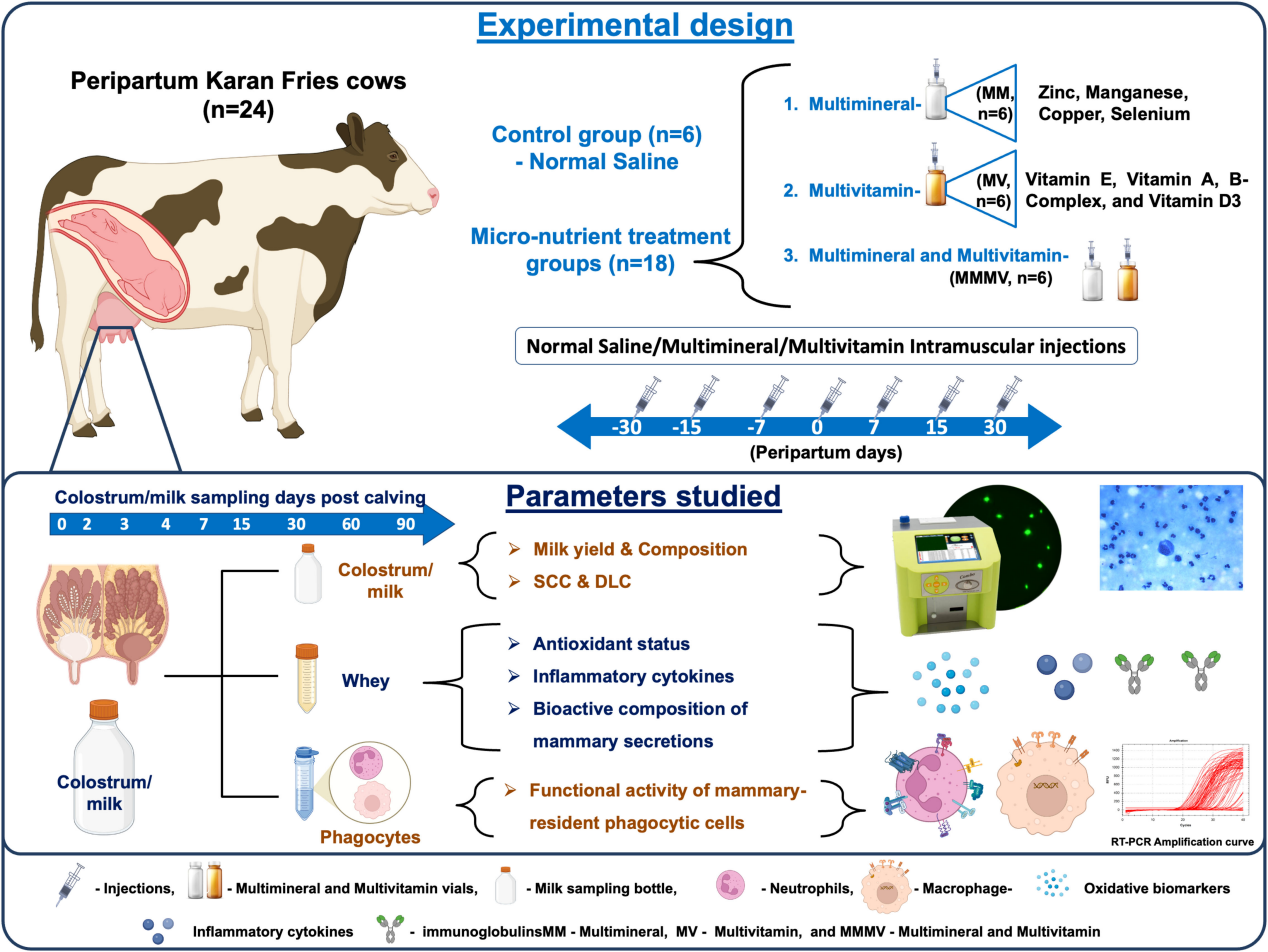
Schematic illustration of the experimental design showing the treatment of transition dairy cows, the timing of sample collection, and the parameters measured.

To meet the nutritional requirements of the transition period, all cows were fed individually with a total mixed ration (TMR), formulated in accordance with the standard feeding protocols of the institute for transition cows. Detailed information on the chemical composition, premix, and feed ingredients is provided in **Supplementary Table 1**. The control group received only the basal TMR diet and was administered intramuscular injections of sterile normal saline (5 ml). The MM group was administered 5 ml of a multimineral injection (Zn 40 mg/ml, Mn 10 mg/ml, Cu 15 mg/ml, Se 5 mg/ml; Stimvet Chelated Multimineral, Wellcon Animal Health, India); the MV group received 5 ml of a multivitamin injection (Vitamin A (1000 IU), Vitamin D_3_ (500 IU), Vitamin E acetate (5 mg), and B-complex vitamins including Niacinamide (10 mg), Thiamine HCl (10 mg), Pyridoxine HCl (5 mg), Riboflavin (5 mg), Choline chloride (5 mg), D-Panthenol (1 mg), D-Biotin (10 µg), and Vitamin B₁₂ (10 µg).; Zenex Ah HIVIT Plus, India); and the MMMV group received both injections (5 ml each). All injections were administered intramuscularly into the neck muscles on days −30, −15, −7, 0 (day of parturition), +7, +15, and +30 relative to calving (**Figure 1**). The dosing schedule and repeated injection protocol were designed in accordance with manufacturer guidelines and supported by earlier published studies (16, 18, 21, 22).

### 2.4 Schedule of sample collection and processing of the sample

The calving date was estimated using the farm/stock register, with a margin of one week before or after the anticipated calving date, as recorded by the Institute’s Livestock Farm. The colostrum and milk samples, which represent all four quarters, were obtained on the day of calving (day 0) and on days 2, 3, 4, 7, 15, and 30 post-calving. The samples were collected in sterile tubes (200 ml/cow). The milk samples were collected using manual and automated milking methods. Hand milking was employed throughout the colostrum phase, while machine milking was used for the rest of the experiment. All animals remained clinically healthy and did not show any signs of mastitis during the entire study period.

### 2.5 Composition, somatic cell count, and differential leukocyte count of colostrum and milk

Colostrum and milk composition, i.e., fat, protein, lactose, and solids not fat (SNF), were estimated by a lactose milk analyzer (Lactoscan MCCW - V3, Bulgaria) as described by the manufacturer’s instructions. To estimate the somatic cell count (SCC), the samples were examined using the Lactoscan somatic cell counter (Milkotronic Ltd, Stara Zagora, Bulgaria) as described by Alhussien and Dang (23). The differential leukocyte count (DLC) of colostrum and milk was performed to examine the percentage of phagocytic cells released in the milk, which includes neutrophils, macrophages, and the ratio of neutrophils to macrophages. The DLC was estimated using the method described by Dang et al. (24).

### 2.6 Isolation and phagocytic activity of colostrum and milk phagocytes

Neutrophils and macrophages were isolated from colostrum and milk samples using gradient density centrifugation as described by Alhussien et al. (3). The functional activity of these cells was evaluated *in vitro* by measuring their phagocytic activity (PA) with the nitro blue tetrazolium (NBT) reduction assay, following the protocol of Alhussien et al. (3).

### 2.7 Gene expression analysis of milk phagocytes

According to the manufacturer’s instructions, total RNA from milk phagocytes was extracted and purified using the TRIzol reagent (Invitrogen, Carlsbad, CA). To remove the genomic contamination, DNase Set (Qiagen, India Pvt Ltd.) was used as per the manufacturer’s protocol. RNA integrity was evaluated by agarose gel electrophoresis (1.8% agarose), and the RNA quality and quantity were verified by taking the absorption reading at λ_260_/λ_280_ using the Bio Spec-nano (Serial No A116449; Biotech). For the preparation of cDNA, 1µg of total RNA was reverse transcribed using the Verso cDNA synthesis kit (Thermo Scientific, USA) as per the manufacturer’s instructions. The primers selected from the published literature are provided in the **Supplementary Table 2** (3, 25). The primers were optimized by gradient PCR for the annealing temperature of each primer for specific bovine chemokine receptors (CXCR1 and CXCR2), glucocorticoid receptor (GR-α), toll-like receptors (TLR2, TLR4), cluster of differentiation (CD25), and the endogenous genes (GAPDH (glyceraldehyde-3-phosphate dehydrogenase) and β-actin (beta-actin)). The PCR products were subjected to agarose gel electrophoresis (1.8%) to visualize the PCR amplified product size at a specific size. Following the PCR optimization, quantitative real-time PCR (qPCR) (Roche’s Light cycler 480) was done using the SYBR Green (Thermo Scientific, USA) according to the manufacturer’s protocol. Briefly, a total reaction volume of 10 µl was prepared by combining 1 µl of cDNA, 5 µl of SYBR green (2x) mixes, and 0.5 µl each of forward and reverse primers. Nuclease-free water was used to make up the remaining volume. The reaction of qPCR consisted of initial heating at 50 °C for 2 minutes and 95 °C for 10 minutes, and the contents were amplified for 40 cycles (95 °C for 30s, 59 °C for all genes for 30s). The final extension was done at 72 °C for 10 minutes. GAPDH and β-actin were used as reference genes for normalization in the gene expression analysis, and the mRNA abundance on the day of calving (day 0) of the control group was taken as a calibrator with which the relative expression of all groups during different time points was estimated. The 2^-ΔΔCt^ method was used to assess the relative quantification of all genes (26).

### 2.8 Isolation of colostrum/milk whey

Colostrum and milk samples were centrifuged at 4000 × g for 30 minutes at 4 °C to eliminate the fat layer. To the defatted colostrum/milk, rennet (0.25 μg/ml; Sigma, Missouri, USA) was added to induce casein precipitation. The mixture was gently stirred multiple times and incubated at 37 °C for 30 minutes. Following incubation, a second centrifugation was carried out at 3000 × g for 10 minutes to obtain the supernatant (whey), which was subsequently stored in 10 ml tubes at −20 °C for further analysis.

### 2.9 Quantification of oxidative stress biomarkers

The concentration of total antioxidant capacity (TAC) in colostrum and milk whey samples was measured using the QuantiChrom™ assay kit (BioAssay Systems, Hayward, CA, USA). The detection range for TAC was between 0.0015 and 1 mmol/L (DTAC-100). The intra-assay and inter-assay coefficients of variation were below 8% and 10%, respectively. Additionally, bovine-specific ELISA kits were employed to assess the enzymatic activities of superoxide dismutase (SOD; Wuhan Fine Biotech, Wuhan, China), catalase (CAT; Bioassay Technology Laboratory, Shanghai, China), and glutathione peroxidase (GPx; Cat. No. E0006Bo). The sensitivity of the assays for SOD (Cat. No. EB0164), CAT (Cat. No. E0025Bo), and GPx was 0.469 ng/ml, 0.28 ng/ml, and 0.58 ng/ml, respectively. The standard curve ranges were 0.781–50 ng/ml for SOD, 0.5–200 ng/ml for CAT, and 1–300 ng/ml for GPx. Intra-assay and inter-assay variations were maintained below 8% and 10%, respectively. Optical density (OD) values were recorded using an ELISA plate reader (Multiskan Go, Thermo Scientific, Finland).

### 2.10 Quantification of inflammatory cytokines

Inflammatory cytokines; interleukin-1 alpha (IL-1α), interleukin-1 beta (IL-1β), interleukin-4 (IL-4), interleukin-6 (IL-6), interleukin-8 (IL-8), interleukin-10 (IL-10), interleukin-17A (IL-17A), interferon-gamma (IFN-γ), and tumor necrosis factor-alpha (TNF-α) in colostrum and milk whey. were quantified simultaneously using the MILLIPLEX^®^ Bovine Cytokine/Chemokine Magnetic Bead Panel 1 - Immunology Multiplex Assay Kit (Cat. # BCYT1-33K, Merck Life Sciences, Darmstadt, Germany), which utilizes Luminex^®^ xMAP^®^ technology. Detailed assay characteristics for each cytokine included in the multiplex panel are provided in **Supplementary Table 3**.

### 2.11 Quantification of immunoglobulins and insulin-like growth factors

Immunoglobulin G (IgG) and Immunoglobulin A (IgA) concentrations in colostrum and milk whey were quantified using bovine-specific ELISA kits (Bioassay Technology Laboratory, Shanghai, China). The assay sensitivity limits were 1.03 µg/ml for IgG (Cat. No. E0010Bo) and 0.13 mg/ml for IgA (Cat. No. E0009Bo). The quantification ranges extended from 2 to 600 µg/ml for IgG and 0.2 to 70 mg/ml for IgA. Insulin-like growth factor levels (IGF-I and IGF-II) in colostrum and milk whey were evaluated by using bovine-specific ELISA kits. The IGF-I kit was obtained from Bioassay Technology Laboratory (Shanghai, China), and the IGF-II kit from CUSABIO (Houston, USA). The minimum detectable limits for IGF-I (Cat. No. E0016Bo) and IGF-II (Cat. No. CSB-EL011088BO) were 0.53 ng/ml and 7.81 ng/ml, respectively. The detection ranges were 1–400 ng/ml for IGF-I and 31.25–2000 ng/ml for IGF-II. All ELISA assays demonstrated intra-assay and inter-assay variability below 8% and 10%, respectively. For all these parameters, absorbance was measured using an ELISA microplate reader (Multiskan Go, Thermo Scientific, Finland).

### 2.12 Statistical analysis

The data was analyzed using the SPSS software system (version 22). All the data obtained from the study were expressed as mean ± standard error and were analyzed by two-way ANOVA. Tukey’s multiple comparison test was used to determine statistical significance, which was set at P < 0.05. The effect of the treated groups (Gr), days (D) of the periparturient period, and their interactions (Gr×D) were estimated using the statistical model shown below


$${\text{Y}}_{\text{ijk}}=\text{μ}+\text{ G}{\text{r}}_{\text{i}}+\;{\text{D}}_{\text{j}}+\;\left(\text{GrD}\right)\text{ijj }+\;{\text{e}}_{\text{ijk}}$$

Where, Y_ijk_ is a dependent variable, μ is the overall mean of the population, Gr_i_ is the effect of micronutrient feeding (i = 4), D_j_ is the effect due to the measurement days (j = 7), and (GrD)_ij_ is the effect due to treatment group by measurement days’ interactions, and e_ijk_ is the residual error.

## 3 Results

### 3.1 Milk yield and composition

**Figure 2:**
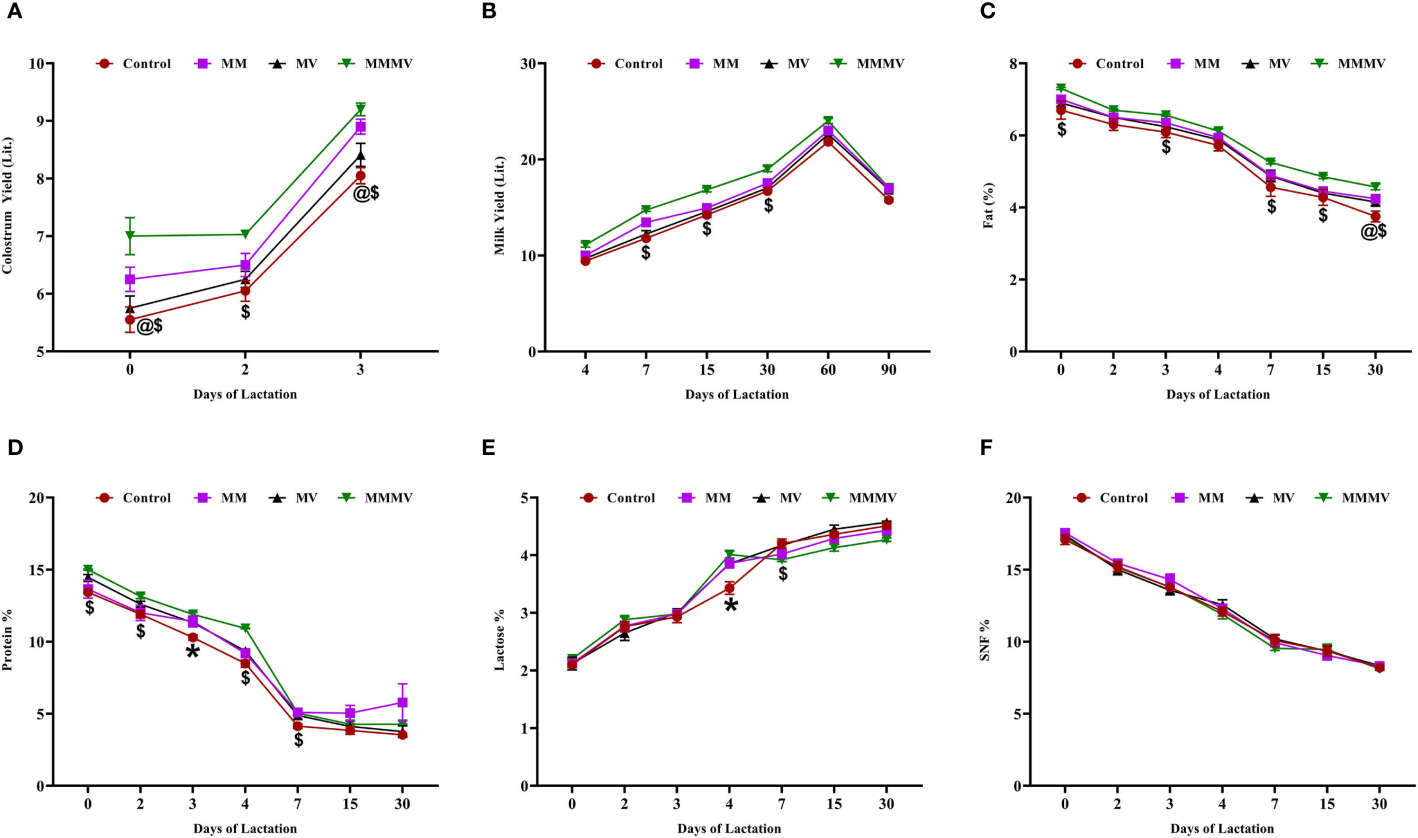
Colostrum and milk yield and composition in control and treatment groups of crossbred cattle, including colostrum yield **(A)**, milk yield **(B)**, fat (%) **(C)**, protein (%) **(D)**, lactose (%) **(E)**, and SNF (%) **(F)**. Control: received basal diet only; MM: basal diet + injectable multiminerals; MV: basal diet + injectable multivitamins; MMMV: basal diet + combination of injectable multiminerals and multivitamins. Mean values with different superscript symbols indicate significant differences compared to the control group: @ control vs MM, $ control vs MMMV, * control vs MM, MV, and MMMV. Differences were considered statistically significant at P < 0.05.

The colostrum and milk yield, as well as the fat and protein percentages, were highest (P < 0.05) in the MMMV group, followed by the multi-mineral, multi-vitamin, and control groups (**Figures 2A–D**). Fat and protein percentages were highest in colostrum samples and showed a consistent decline, reaching their lowest levels by day 30 post-calving. Lactose concentration increased over time, peaking on day 30 post-calving, while the SNF percentage declined, reaching its minimum on the same day. The difference in the percentage of lactose and SNF among the groups was minimal (**Figures 2E, F**).

### 3.2 SCC and DLC

**Figure 3:**
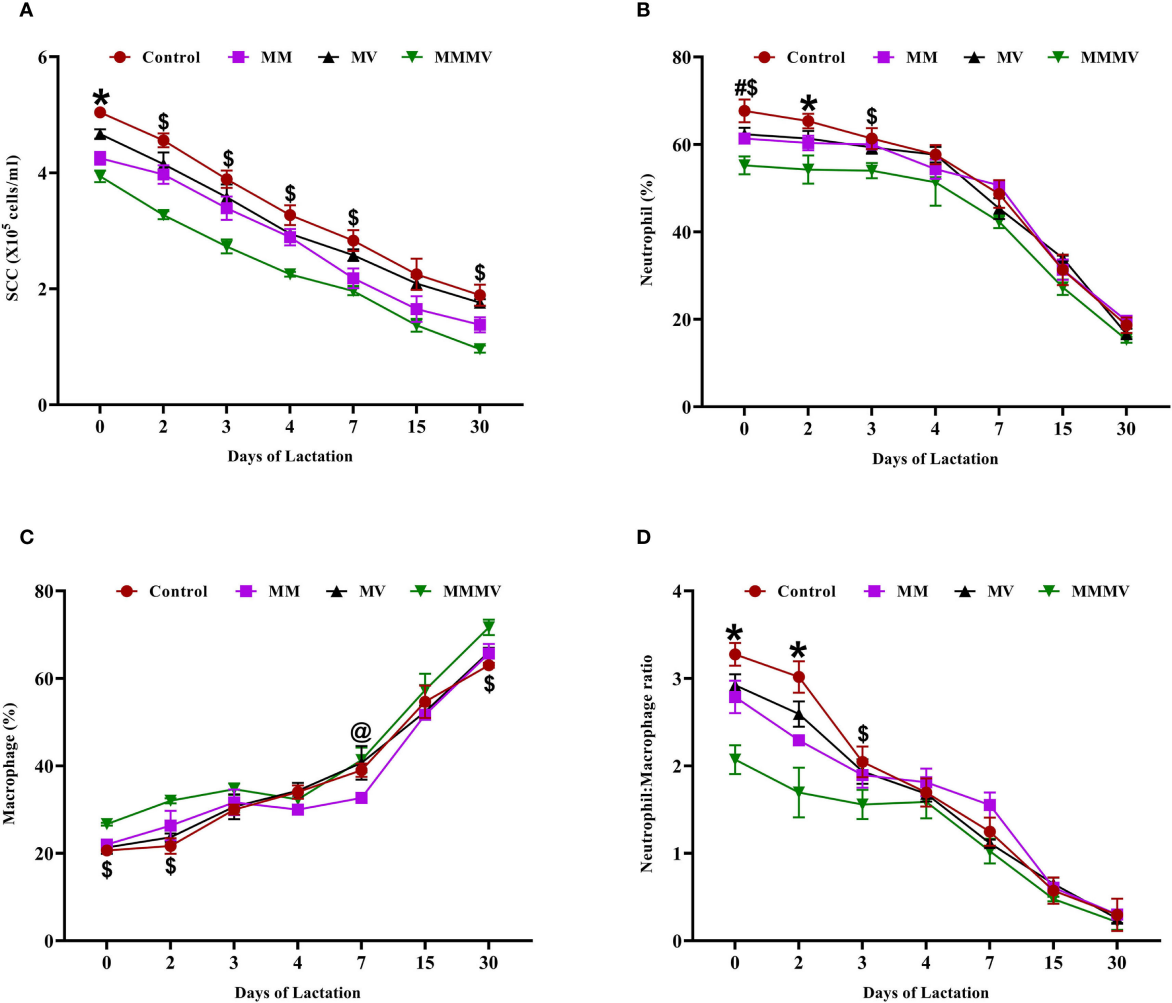
Somatic cell and immune cell composition in milk of control and treatment groups of crossbred cattle, including somatic cell count (SCC; ×10⁵ cells/ml) **(A)**, neutrophil percentage **(B)**, macrophage percentage **(C)**, and neutrophil-to-macrophage ratio **(D)**. Mean values with different superscript symbols indicate significant differences compared to the control group: @ control vs MM, # control vs MV, $ control vs MMMV, * control vs MM, MV, and MMMV. Differences were considered statistically significant at P < 0.05.

Somatic cell count (SCC) was highest at calving across all groups. Colostrum SCC values were significantly higher (P < 0.05) in the control group compared to the treatment groups (**Figure 3A**). Throughout the study period, SCC remained consistently and significantly lower (P < 0.05) in the MMMV group, followed by the MM, MV, and control groups, respectively. Differential leukocyte count (DLC) analysis showed a higher neutrophil percentage and a lower macrophage percentage in the colostrum of the control group compared to the other groups (**Figures 3B, C**). The neutrophil-to-macrophage (N: M) ratio was highest on the day of calving across all groups and declined as lactation progressed. The N: M ratio in colostrum was significantly higher (P < 0.05) in the colostrum of the control group, followed by MV, MM, and MMMV groups, respectively (**Figure 3D**).

### 3.3 Phagocytic activity of neutrophils and macrophages

**Figure 4:**
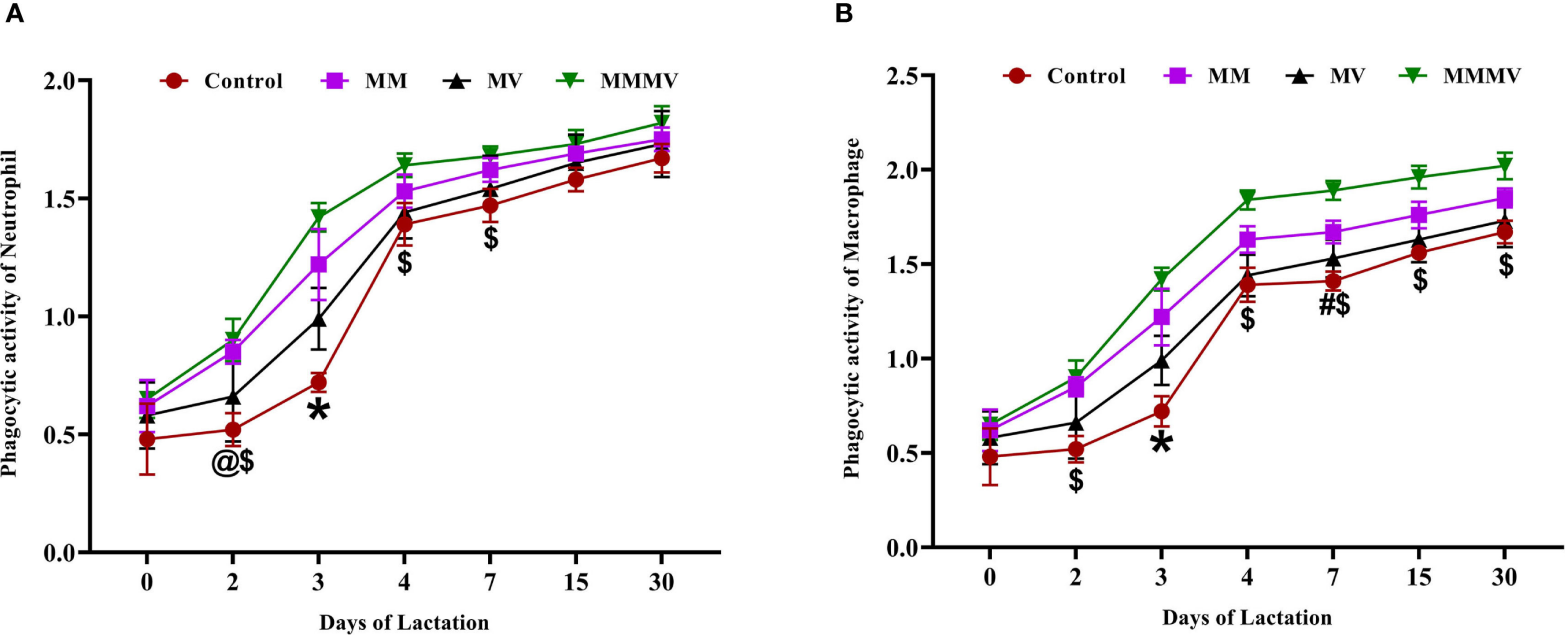
Phagocytic activity of milk immune cells in control and treatment groups of crossbred cattle, including phagocytic activity of neutrophils **(A)** and phagocytic activity of macrophages **(B)**. Mean values with different superscript symbols indicate significant differences compared to the control group: @ control vs MM, # control vs MV, $ control vs MMMV, * control vs MM, MV, and MMMV. Differences were considered statistically significant at P < 0.05.

The PA of both milk neutrophils and macrophages was lowest around parturition (day 0) in all groups and gradually increased throughout the study period, reaching peak levels by day 30 postpartum (**Figure 4**). For neutrophils, the MMMV group consistently exhibited the highest PA, with significant differences (P < 0.05) compared to the control, MM, and MV groups, particularly evident in the colostrum phase (days 0–3). The MM and MV groups also showed enhanced PA compared to the control group, but to a lesser extent than the MMMV group (**Figure 4A**). While MM and MV groups showed improved PA of macrophages relative to controls, the most pronounced and consistent enhancement (P < 0.05) in PA was observed in the MMMV group throughout the entire 30-day lactation period (**Figure 4B**).

### 3.4 Genes and receptor expression of neutrophils and macrophages

**Figure 5:**
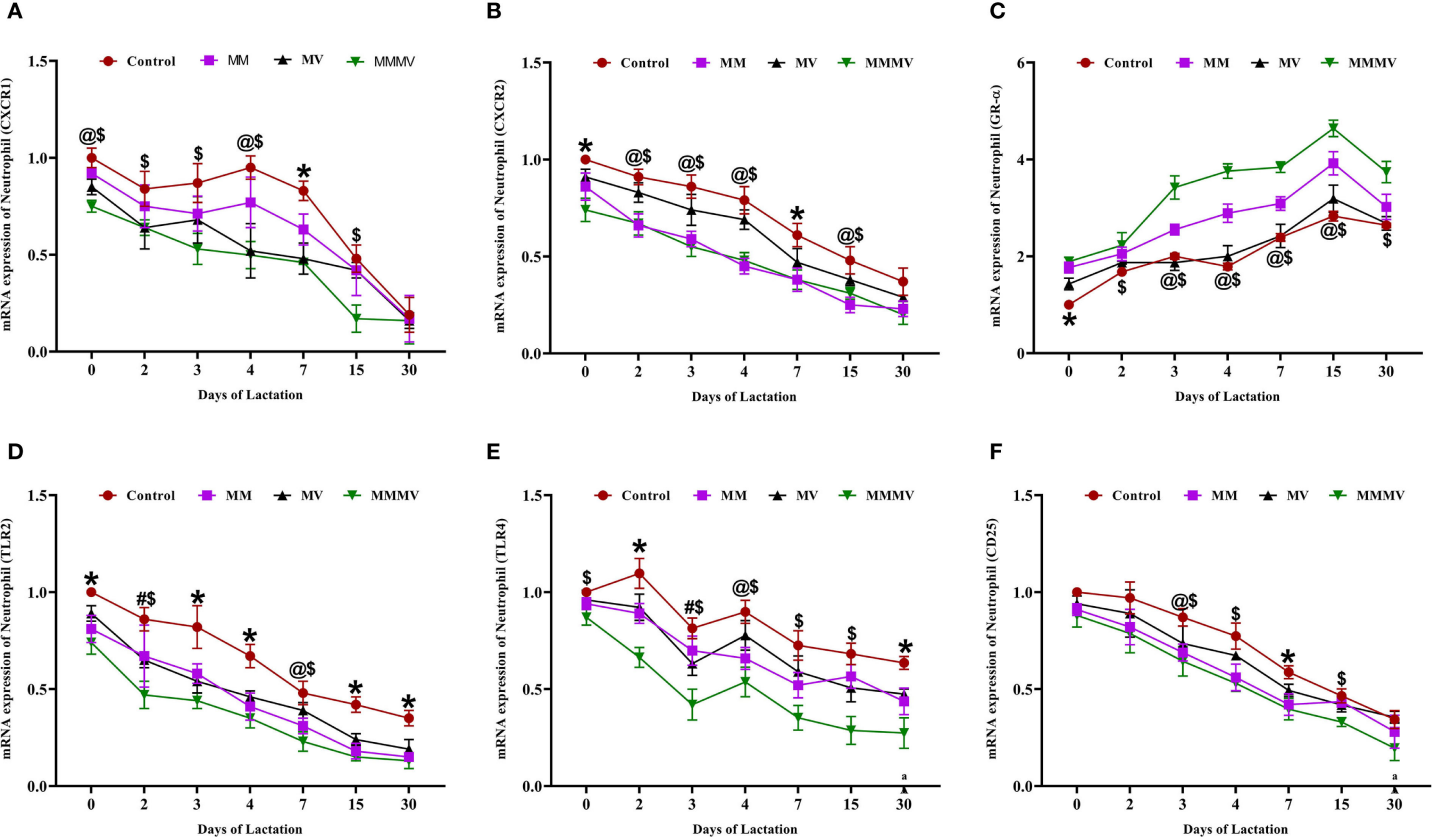
Relative mRNA expression of immune-related genes in milk neutrophils of control and treatment groups of cows, including CXCR1 **(A)**, CXCR2 **(B)**, GR-α **(C)**, TLR2 **(D)**, TLR4 **(E)**, and CD25 **(F)**. Mean values with different superscript symbols indicate significant differences compared to the control group: @ control vs MM, # control vs MV, $ control vs MMMV, * control vs MM, MV, and MMMV. β-actin and GAPDH were used as endogenous genes, and the mRNA abundance on the day of calving (day 0) in the control group was used as the calibrator to estimate the relative expression of all groups across different time points. Differences were considered statistically significant at P < 0.05.

**Figure 6:**
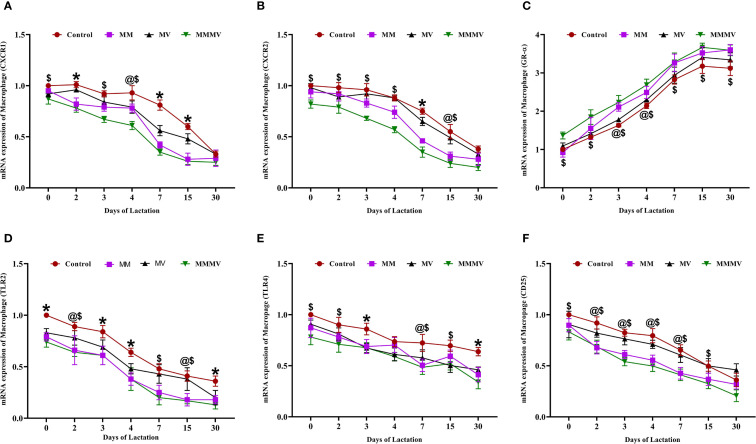
Relative mRNA expression of immune-related genes in milk macrophages of control and treatment groups of cows, including CXCR1 **(A)**, CXCR2 **(B)**, GR-α **(C)**, TLR2 **(D)**, TLR4 **(E)**, and CD25 **(F)**. Mean values with different superscript symbols indicate significant differences compared to the control group: @ control vs MM, $ control vs MMMV, * control vs MM, MV, and MMMV. β-actin and GAPDH were used as endogenous genes, and the mRNA abundance on the day of calving (day 0) in the control group was used as the calibrator to estimate the relative expression of all groups across different time points. Differences were considered statistically significant at P < 0.05.

The mRNA expression of several immune-related genes (CXCR1, CXCR2, TLR2, TLR4, and CD25) followed a similar pattern in both milk neutrophils and macrophages across the lactation period (**Figures 5**, **6**). These markers exhibited the highest expression around parturition (days 0–4) and gradually declined toward day 30 post-calving in all groups. At nearly all time points, the control group maintained significantly higher (P < 0.05) expression levels of these genes compared to the MM, MV, and especially the MMMV group, which showed the most pronounced downregulation throughout lactation. Although the overall trend was consistent across both cell types, the suppression of pro-inflammatory gene expression was more evident in neutrophils, suggesting a stronger response to supplementation in this milk immune population.

In contrast, GR-α (glucocorticoid receptor alpha) expression was lowest around calving and increased gradually postpartum in all the groups. The MMMV group displayed significantly higher (P < 0.05) GR-α expression than all other groups. The control group consistently exhibited the lowest GR-α expression across the study period.

### 3.5 Oxidative stress biomarkers in colostrum and milk

**Figure 7:**
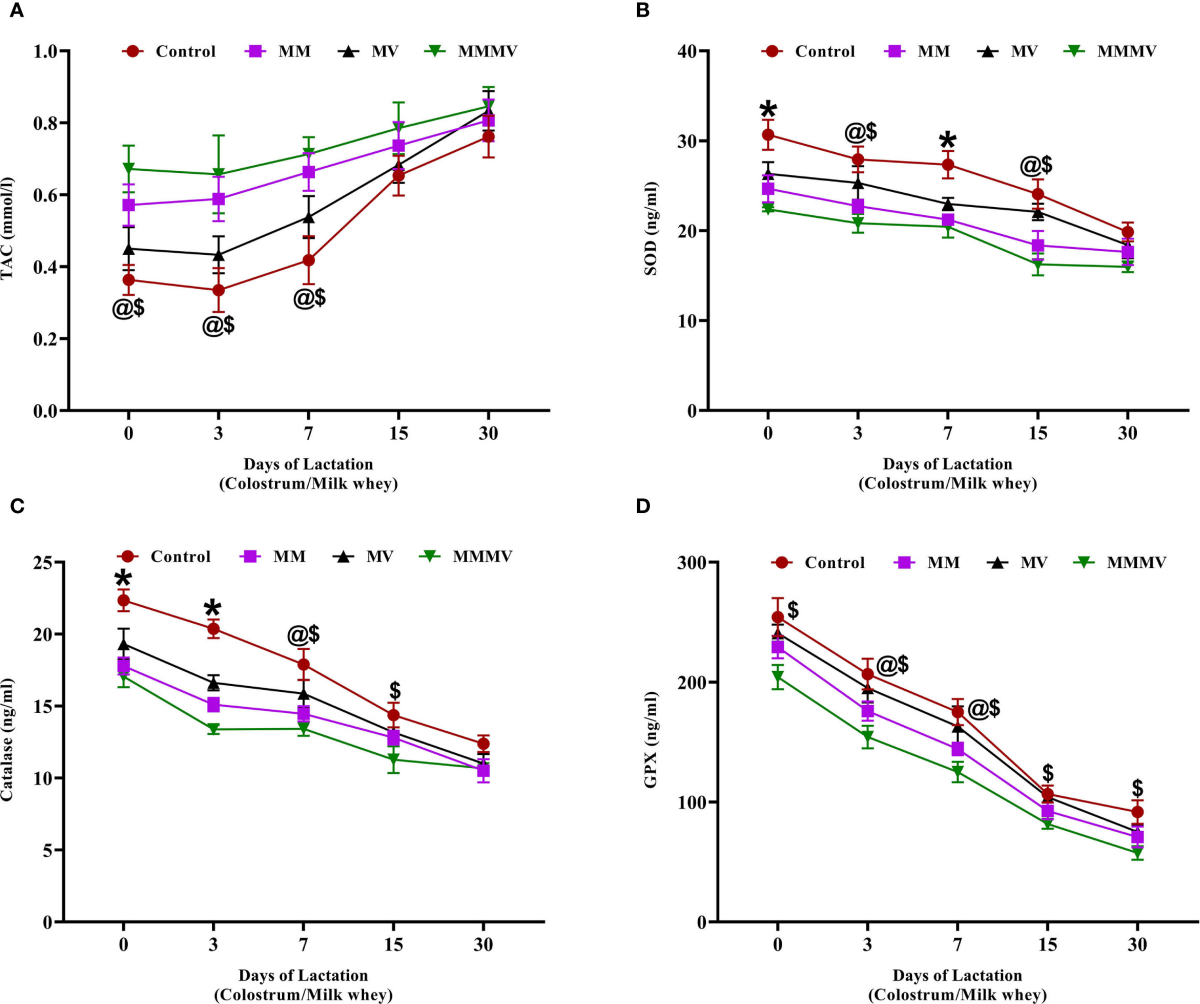
Milk whey concentration of oxidative stress biomarkers in control and treatment groups of cows, including total antioxidant capacity (TAC, mmol/L) **(A)**, superoxide dismutase (SOD, ng/ml) **(B)**, catalase (CAT, ng/ml) **(C)**, and glutathione peroxidase (GPX, ng/ml) **(D)**. Mean values with different superscript symbols indicate significant differences compared to the control group: @ control vs MM, $ control vs MMMV, * control vs MM, MV, and MMMV. Differences were considered statistically significant at P < 0.05.

Oxidative stress markers, including total antioxidant capacity (TAC), SOD, CAT, and GPx, showed distinct temporal trends from day 0 to day 30 post-calving across all groups (**Figure 7**). TAC was lowest at calving in all groups and increased progressively over time. The MMMV group consistently exhibited the highest TAC values, followed by MM, MV, and control groups, respectively, with the differences between groups remaining relatively stable across the study period (**Figure 7A**). In contrast, the enzymatic antioxidants (SOD, CAT, and GPx) declined steadily from day 0 to day 30. Their activities were highest in the control group and progressively lower in the MV, MM, and MMMV groups (P < 0.05) (**Figures 7B–D**). The inverse relationship between TAC and enzyme activity suggests that cows receiving MMMV supplementation experienced lower oxidative stress and thus required less activation of endogenous antioxidant enzymes.

### 3.6 Pro- and anti-inflammatory cytokines in colostrum and milk whey

#### 3.6.1 Pro-inflammatory cytokine

**Figure 8:**
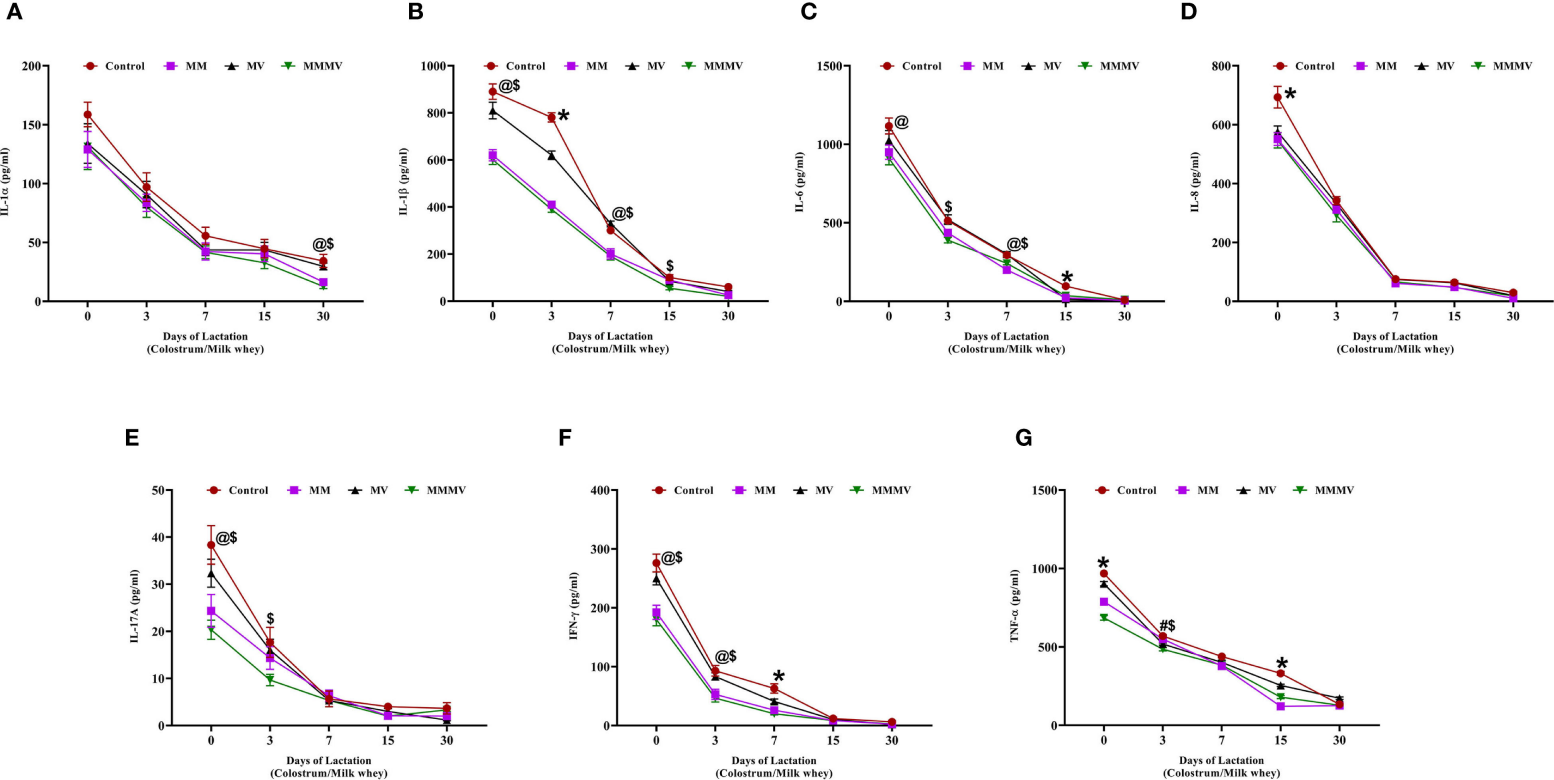
Milk whey concentration of pro-inflammatory cytokines (pg/mL) in control and treatment groups of cows, including IL-1α **(A)**, IL-1β **(B)**, IL-6 **(C)**, IL-8 **(D)**, IL-17A **(E)**, IFN-γ **(F)**, and TNF-α **(G)**. Mean values with different superscript symbols indicate significant differences compared to the control group: @ control vs MM, # control vs MV, $ control vs MMMV, * control vs MM, MV, and MMMV. Differences were considered statistically significant at P < 0.05.

All pro-inflammatory cytokines, including IL-1α, IL-1β, IL-6, IL-8, TNF-α, IFN-γ, and IL-17A, were highest on the day of calving and declined steadily throughout the 30-day post-calving period in all groups (**Figure 8**). The control group consistently exhibited higher pro-inflammatory cytokine levels, particularly during the colostrum phase. In contrast, cows supplemented with MMMV showed significantly reduced levels of IL-1β, IL-6, TNF-α, and IFN-γ across the entire 30-day period (P < 0.05). These reductions were most prominent at early time points but persisted through day 30. IL-8 and IL-17A levels showed a significant group difference only on day 0, with higher concentrations in the control group. IL-1α, while following a similar downward trend, did not exhibit consistent differences between groups across time points.

#### 3.6.2 Anti-inflammatory cytokines

**Figure 9:**
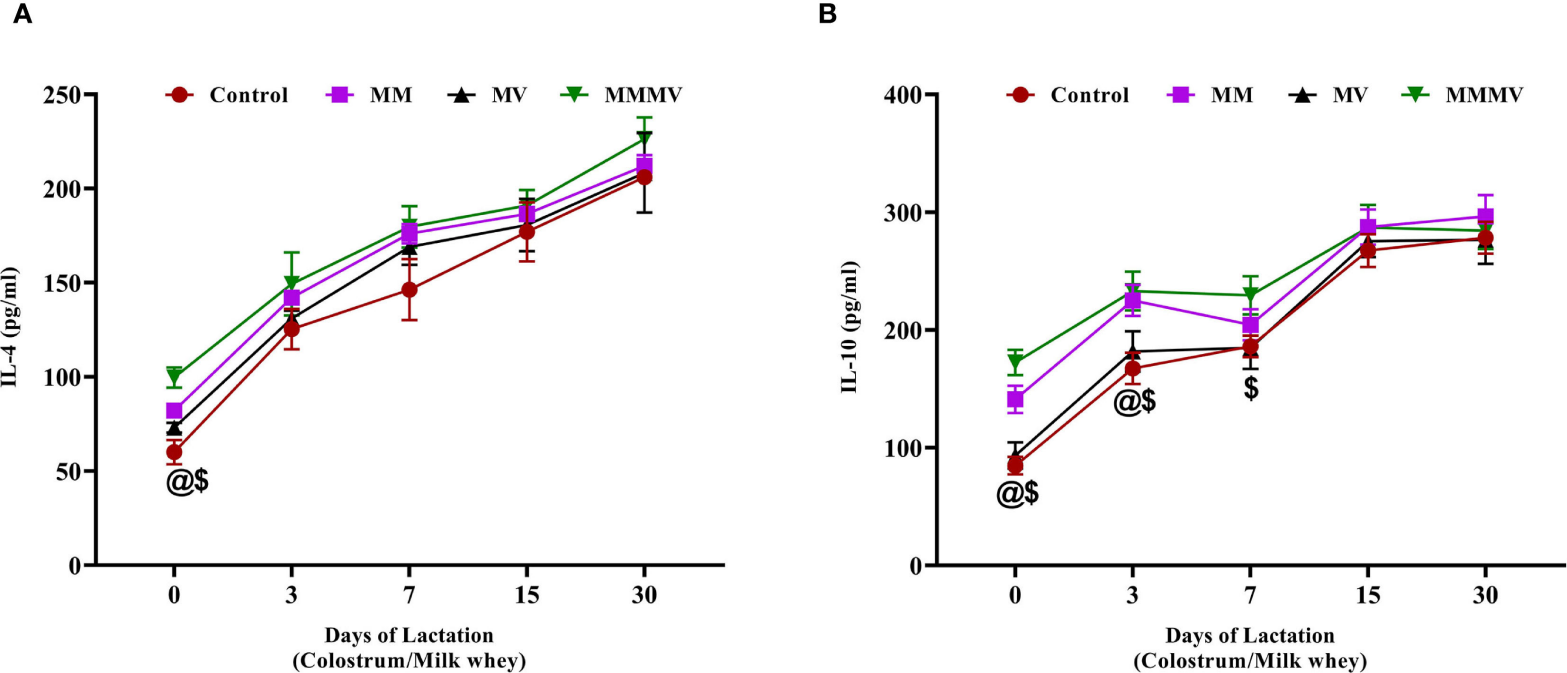
Milk whey concentration of anti-inflammatory cytokines (pg/ml), including IL-4 **(A)**, and IL-10 **(B)**. Mean values with different superscript symbols indicate significant differences compared to the control group: @ control vs MM, $ control vs MMMV. Differences were considered statistically significant at P < 0.05.

The concentrations of IL-4 and IL-10 in milk whey increased steadily throughout the lactation period in all groups (**Figure 9**). On the day of calving (day 0), both cytokines were significantly lower (P < 0.05) in the control group compared to the supplemented groups, with the MMMV group showing the highest levels. For IL-4, this difference was statistically significant only at day 0, with levels converging among all groups by day 3 and remaining similar through day 30. In contrast, IL-10 concentrations remained significantly higher (P < 0.05) in the MMMV group compared to other groups from day 0 through day 7, indicating a more sustained anti-inflammatory effect.

### 3.7 Immunoglobulins and insulin-like growth factors

**Figure 10:**
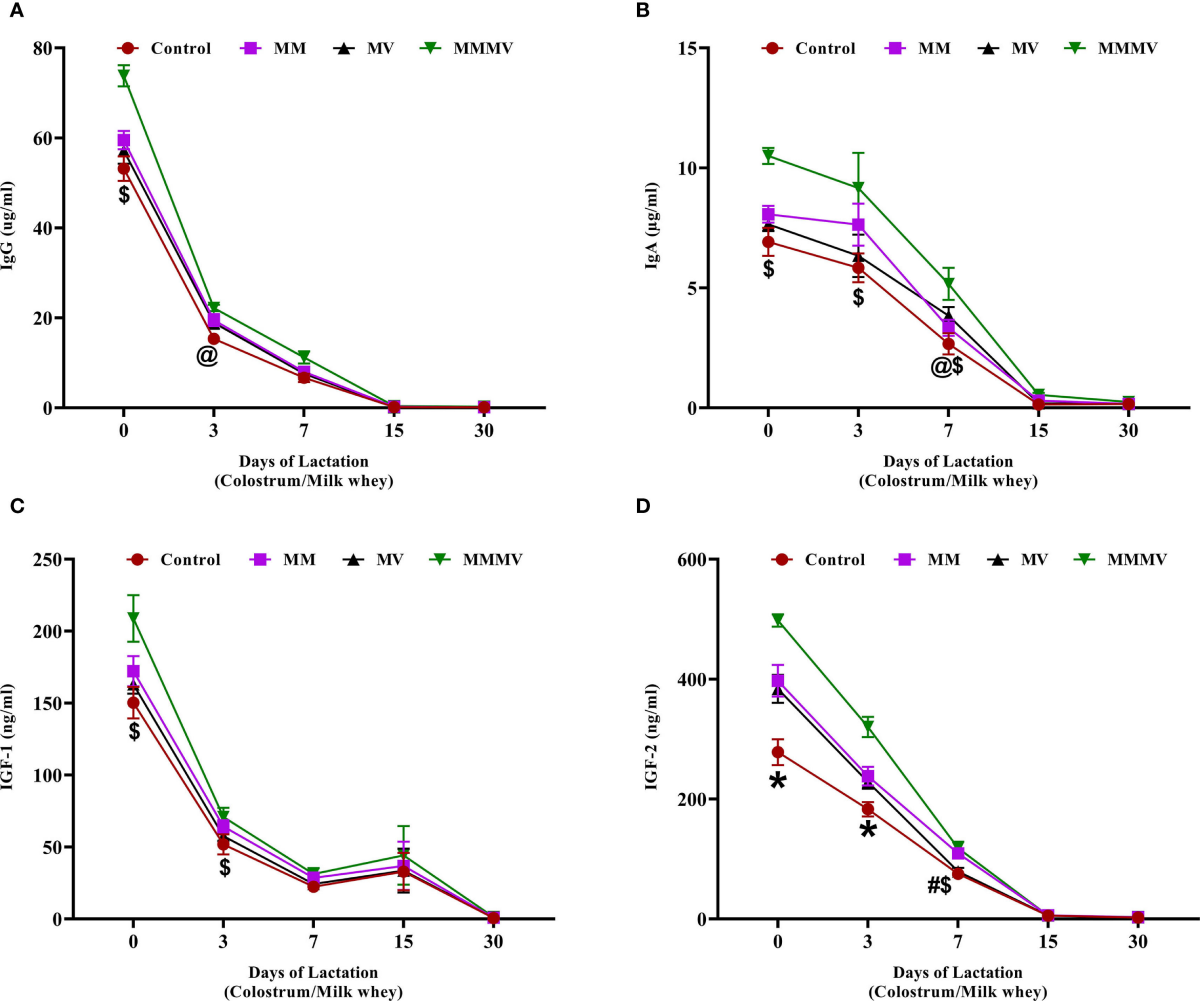
Milk whey concentration of immunoglobulins and growth factors in control and treatment groups of cows, including IgG (µg/ml) **(A)**, IgA (µg/ml) **(B)**, IGF-1 (ng/ml) **(C)**, and IGF-2 (ng/ml) **(D)**. Mean values with different superscript symbols indicate significant differences compared to the control group: @ control vs MM, # control vs MV, $ control vs MMMV, * control vs MM, MV, and MMMV. Differences were considered statistically significant at P < 0.05.

The concentrations of immunoglobulins (IgG and IgA) and insulin-like growth factors (IGF-1 and IGF-2) in milk whey were highest on the day of calving and declined progressively throughout the study period. IgG and IgA (**Figures 10A, B**) peaked at calving and showed a sharp decline by day 7. Cows in the MMMV group exhibited significantly higher (P < 0.05) IgG and IgA concentrations on days 0 and 3 compared to the control group. By day 15, immunoglobulin levels had decreased substantially and were similar across all groups. IGF-1 and IGF-2 (**Figures 10C, D**) followed a similar declining trend over time. On days 0 and 3, IGF-1 concentrations were significantly higher (P < 0.05) in the MMMV group compared to other groups. The effect of supplementation was even more pronounced for IGF-2. On days 0 and 3, IGF-2 levels were higher (P < 0.05) in the MMMV group than in the control, while MM and MV showed intermediate concentrations. By day 15, IGF-2 levels in all groups had dropped to near-baseline and remained low through day 30.

## 4 Discussion

Recent research underscores the benefits of parenteral micronutrient administration as an efficient method for delivering essential nutrients directly to target tissues (11, 27, 28). This approach can help maintain physiological functions and support a balanced immune response, significantly impacting health during the transition period. Our study focused on the effects of parenteral micronutrient supplementation on mammary gland immune defense mechanisms and its potential to enhance colostrum and milk quality.

Micronutrients such as copper, iodine, and B vitamins are crucial for protein synthesis and metabolic functions. Selenium, for example, enhances antioxidant mediators and upregulates genes associated with milk protein synthesis (29). Our results showed a significant increase in fat and protein percentages in colostrum, transitional milk, and milk samples, with the highest percentages observed in cows receiving combined mineral and vitamin supplementation. This aligns with previous studies, such as those by Griffiths et al. (30) and Kay et al. (31), which reported increased fat content in colostrum and milk following supplementation with trace minerals and vitamins. Lactose synthesis in the mammary gland is influenced by micronutrients like manganese, a cofactor for enzymes involved in carbohydrate metabolism (32). Chawla and Kaur (33) found that supplementation with vitamins E and A increases milk yield, which our study also confirmed. Interestingly, our findings show that cows receiving combined mineral and vitamin supplementation produced colostrum with significantly higher IGF-1 and IGF-2 levels during the first three days after calving, an increase that likely promotes greater metabolic activity and directs nutrients toward early milk production. Prior studies show that vitamin D_3_ administration enhances insulin sensitivity, energy metabolism, and hepatic IGF-1 synthesis, thereby promoting its transfer to the mammary gland (34, 35). In addition, micronutrient-driven reductions in mammary inflammatory cytokines and oxidative stress factors known to suppress IGF-1 during the transition period (36–38) provide a mechanistic basis for the elevated IGF levels and greater milk yield we recorded. Elevated colostral immunoglobulins and insulin-like growth factors (IGFs) in the MMMV group not only reflect improved maternal mammary health but also indicate enhanced passive immune transfer, which is critical for neonatal resilience. Injectable maternal micronutrient supplementation has been shown to increase colostral immunoglobulin content and positively modulate neonatal oxidative status (39).

SCC is a reliable milk quality measure, influenced by factors like milk production, season, and mammary inflammation (23, 40). Our research found SCC highest at calving, with the control group showing higher colostrum SCC than the treated groups. Zinc supplementation reduces mastitis by promoting skin health and integrity, supporting keratin lining formation in the teat canal, which acts as a barrier against bacterial entry (41). Organic forms of zinc, copper, and selenium decrease SCC in early lactation (42). Vitamins A and E are crucial for udder health, reducing SCC by mitigating oxidative stress and maintaining mammary epithelial cell integrity. Trace minerals and vitamins act as powerful modulators of immune signaling and antioxidant defense, as demonstrated by their ability to reduce somatic cell counts, attenuate inflammatory markers, and restore redox balance in dairy cattle during the periparturient phase. Recent studies have further confirmed these benefits, highlighting the role of injectable micronutrient supplementation in enhancing immune function and oxidative status in dairy cattle (11, 18, 39). Collectively, our data indicate that this comprehensive nutritional strategy optimizes the interplay among mineral homeostasis, vitamin metabolism, and growth-factor signaling, culminating in improved colostrum quality and subsequent milk production.

Milk is rich in immune cells, including phagocytic cells (neutrophils and macrophages), essential for maintaining udder health by combating infections and ensuring tissue homeostasis (43). Macrophages serve as the predominant sentinels in the normal mammary gland, acting as the first line of immune surveillance against invading mastitis-causing pathogens. Upon pathogen detection, activated macrophages release chemoattractants that facilitate neutrophil migration from blood to infected tissue, establishing a coordinated inflammatory response (20, 44). Micronutrients play a vital role in supporting the function of these immune cells and reducing oxidative stress, which is crucial during the periparturient period when cows are more vulnerable to diseases due to physiological stress (29, 45). The enhanced phagocytic and oxidative burst activities observed in our study reflect the essential roles of selenium, copper, and zinc as cofactors for key enzymes involved in neutrophil and macrophage bactericidal functions (18).

In the present study, the phagocytic activity of both milk neutrophils and macrophages peaked around parturition and was significantly enhanced in the combined supplemented group, followed by the multiminerals, multivitamins, and control groups. This increase in phagocytic function was paralleled by higher milk immunoglobulin concentrations, elevated total antioxidant capacity, and reduced levels of endogenous antioxidant enzymes such as superoxide dismutase and catalase, indicating diminished oxidative burden in the MMMV group. These findings suggest that parenteral micronutrient supplementation during the transition period supports mammary immune competency and reduces oxidative stress, thereby promoting better colostrum quality and mammary tissue integrity. The observed positive correlation between phagocytic cell activity and immunoglobulin secretion aligns with earlier studies demonstrating that improved immune cell function supports immunoglobulin transfer into milk (20, 46).

Consistent with previous work, trace element supplementation, particularly zinc, copper, and manganese, has been shown to enhance neutrophil oxidative burst and macrophage-mediated antigen presentation, while also improving milk immunological and biochemical properties (7, 45, 47). Notably, control cows in our study exhibited a higher proportion of neutrophils and a lower percentage of macrophages in milk, a pattern typically associated with higher inflammatory or stress stimulus (23). Moreover, we observed that mRNA expression profiles of milk-derived macrophages and neutrophils from control cows revealed increased transcription of TLRs, glucocorticoid and pro-inflammatory chemokine receptors, suggesting that in the absence of adequate antioxidant support, mammary tissues experience greater immune activation and cellular stress. The balanced immune response in the combined supplemented group likely reduces mammary tissue damage and may help prevent subclinical mammary inflammation, particularly during the high-risk periparturient window.

Inflammatory cytokines are central to the regulation of mammary gland health, especially during the transition period when cows are more susceptible to infection and metabolic stress. The balance between pro- and anti-inflammatory cytokines is essential for orchestrating immune responses, tissue remodeling, and defense against pathogens (48). Interestingly, our current findings on cytokine modulation in milk mirror the systemic effects we previously reported (20), indicating that repeated micronutrient injections during the transition period also alter inflammatory profiles within the mammary gland. Specifically, we observed a marked reduction in pro-inflammatory cytokines (IL-1β, IL-6, IL-8, IL-17A, TNF-α, and IFN-γ) alongside an enhancement in anti-inflammatory cytokines (IL-4, IL-10) in milk, indicating that the immunomodulatory effects of vitamins and trace minerals extend beyond systemic immunity to local mammary tissue. This modulation may contribute to improved udder health, reduced risk of mastitis, and better postpartum recovery. Several studies have demonstrated that injectable micronutrient supplementation can mitigate tissue-level inflammation and enhance immunity in cattle, supporting our current findings. Zhang et al. (49) reported that vitamin D_3_, through its interaction with vitamin D receptors expressed on immune cells, downregulates pro-inflammatory cytokine production while enhancing macrophage antibacterial activity. For instance, Bittar et al. (50) found that injectable trace minerals, when combined with vaccination, significantly reduced respiratory tissue inflammation and improved immune markers in dairy calves following viral challenge. Pate and Cardoso (51) administered trace minerals, including selenium, copper, zinc, and manganese, via injection and observed decreased hepatic expression of inflammatory markers during aflatoxin challenge, attributing the effects to enhanced antioxidant enzyme activity and reduced oxidative damage in liver tissues. Khan et al. (11) reviewed that injectable interventions, including vitamins (A, D, E) and trace minerals, play a role in dampening NF-κB-mediated pro-inflammatory signaling and strengthening antioxidant defenses. Furthermore, Hong et al. (52) demonstrated that injectable trace mineral supplementation in feedlot cattle reduced pulmonary and systemic inflammation following Mannheimia hemolytica infection, likely through modulation of leukocyte function and cytokine responses. Collectively, these findings underscore the capacity of injectable micronutrients to exert localized anti-inflammatory effects in addition to systemic benefits, primarily through immunomodulatory and antioxidative mechanisms.

## 5 Conclusions

This study demonstrates that parenteral supplementation with a combination of trace minerals and vitamins during the transition period significantly enhances mammary gland immune function, antioxidant capacity, and colostrum and milk quality in dairy cows. The improved nutrient profile, notably the increased levels of IGF, immunoglobulins, and milk components (fat, protein), was accompanied by reduced SCC and decreased concentration of pro-inflammatory cytokines. These changes reflect enhanced phagocytic activity of milk leukocytes and a more balanced immune response within the mammary gland. Notably, the synergistic effects of trace minerals and vitamins support both local and systemic immunity, reduce oxidative stress, and promote tissue integrity during a physiologically vulnerable period. These findings underscore the value of targeted injectable micronutrient strategies to optimize udder health, milk productivity, and postpartum recovery in dairy herds.

## Data Availability

The original contributions presented in the study are included in the article/**Supplementary Material**. Further inquiries can be directed to the corresponding author.
