# Supplementary material for: Parenteral micronutrient supplementation enhances mammary immune function and colostrum–milk quality by modulating cytokine profiles and oxidative stress in transition crossbred cows
Source: Front Immunol. 2025 Sep 12;16:1669246. doi: 10.3389/fimmu.2025.1669246 (PMC12463632; doi:10.3389/fimmu.2025.1669246)
Supplement: Supplementary file 1 [file DataSheet1.docx]

**Supplementary Table 1**. Attributes and chemical composition of total mixed ration (TMR) fed during the experimental period

| **Attributes** | **Content (g/kg DM unless it is mentioned)** |
| --- | --- |
| Berseem fodder | 210 |
| Wheat straw | 197 |
| Ground yellow maize | 277 |
| Groundnut cake | 152 |
| Wheat bran | 64 |
| Rice bran | 87 |
| Mineral mixture and vitamins premix | 10 |
| Salt | 3 |
| **Chemical composition** | |
| Dry matter | 756 |
| Organic matter | 856 |
| Crude protein | 175 |
| Crude fibre | 291 |
| Total ash | 95 |
| NDF, neutral detergent fibre | 386 |
| ADF, acid detergent fibre | 259 |
| Calcium | 10 |
| Phosphorus | 4.1 |
| Magnesium | 2.3 |
| Manganese | 63.83 mg/kg |
| Copper | 13.22 mg/kg |
| Selenium | 0.36 mg/kg |
| Zinc | 49.00 mg/kg |
| Iron | 50 mg/kg |
| Sulfur | 1.69 g/kg |
| Molybdenum | 1.18 mg/kg |
| β-carotene | 51.43 IU/kg |
| DL-alpha-tocopheryl acetate | 60.50 IU/kg |

Premix composition per kilogram: Vitamin A 7,50,000 IU, Vitamin D3 220,000 IU, Vitamin E 5000 mg, Cobalt 150 mg, Copper 1200 mg, Iodine 325 mg, Iron 1500 mg, Selenium 54 mg, Magnesium 6000 mg, Manganese 1500 mg, Zinc 9600 mg, DL-Methionine 1000 mg, Calcium 25.50 %, Phosphorus 12.75 %. Main ionic components: negative dietary cation-anion difference (DCAD) was -24 mEq/100 g of DM during for prepartum period (0.55 % Na, 1.48 % K, 0.61 % Cl, 0.31 % S). The following formula was used to calculate DCAD = [(mEq of K) + (mEq of Na)] – [(mEq of Cl) + (mEq of S)].

**Supplementary Table 2:** Details of various primers used in the study.

| **Genes** | **Sequence (5′→3′)** | **Accession. No.** | **Size (bp)** | **Annealing**  **Temp (**º**C)** |
| --- | --- | --- | --- | --- |
| **CXCR1** | F: AGTCCCCGTGAGATAAGCAC  R: CCAGGTTCAGCAGGTAGACA | EF597244.2 | 163 | 59 |
| **CXCR2** | F: CAACACTGA-CCTGCCCTCTA  R: CCAGGTTCAGCAGGTAGACA | DQ328664.1 | 197 | 59 |
| **GR-α** | F: TGTGGTTTAAAGAGGGCCAAG  R: TTCTACGTTCCCATCACTGAAAAG | XM_024993840.1 | 74 | 58 |
| **TLR2** | F: CTGGCAAGTGGATTATCGACAA  R: TACTTGCACCACTCGCTCTTCA | XM_005217446.4 | 103 | 59 |
| **TLR4** | F:TGCGTACAGGTTGTTCCTAACATT R:TAGTTAAAGCTCAGGTCCAGCATCT | NM_174198.6 | 110 | 59 |
| **CD25** | F: ACATCGGCAGTGGTCTCAG  R: GAACCTCCACATCAGCAAGC | NM_174358.2 | 60 | 58 |
| **GAPDH** | F: GGGTCATCATCTCTGCACCT  R: GGTCATAAGTCCCTCCACGA | NM_001034034 | 176 | 59 |
| **β-actin** | F: ACAGTCCGCCTAGAAGCA  R: TGGCACCCAGCACAATGAAGATC | BT030480.1 | 179 | 58 |

F, Forward; R, Reverse; CXCR-1 and CXCR-2, Chemokine receptors; GRα, Glucocorticoid receptor; TLR2 and TLR4, Toll like receptors and CD25, Cluster of designation molecules; GAPDH and β-actin, housekeeping genes.

**Supplementary Table 3.** Assay characteristics for all cytokines of MILLIPLEX® bovine cytokine immunology multiplex assay (Cat. # BCYT1-33K).

| **Cytokine** | **Accuracy** | **Sensitivity** | **Standard Curve Range** | **Intra-**  **assay %CV** | **Inter-**  **assay %CV** |
| --- | --- | --- | --- | --- | --- |
| **IL-1α** | 94% | 0.36 | 0.3 - 5,000 pg/ml | <10% | <10% |
| **IL-1β** | 91% | 4.93 | 2.6 - 40,000 pg/ml | <10% | <10% |
| **IL-6** | 95% | 11.23 | 2.6 - 40,000 pg/ml | <10% | <15% |
| **IL-8** | 95% | 22.6 | 2.2 - 35,000 pg/ml | <10% | <10% |
| **IL-17A** | 103% | 0.67 | 0.6 - 10,000 pg/ml | <10% | <10% |
| **IFN-γ** | 94% | 0.08 | 0.1 - 2,000 pg/ml | <10% | <10% |
| **TNF-α** | 92% | 22.62 | 12.8 - 200,000 pg/ml | <10% | <15% |
| **IL-4** | 93% | 16.57 | 12.8 - 200,000 pg/ml | <10% | <10% |
| **IL-10** | 96% | 1.05 | 0.96 - 15,000 pg/ml | <10% | <10% |

IL, Interleukin; IFN-γ, Interferon gamma; TNF-α, Tumour necrosis factor.
